# Double Free Style Perforator Propeller Flaps for Large Posterior Trunk Defects Post Sarcoma Excision

**DOI:** 10.1055/s-0045-1808095

**Published:** 2025-05-05

**Authors:** Ameya Bindu, Quazi Ghazwan Ahmad, Dushyant Jaiswal, Vijayendra Gour, Prabha Yadav, Saumya Mathews, Vineet Kumar, Mayur Mantri, Vinay Kant Shankhdhar

**Affiliations:** 1 Department of Plastic and Reconstructive Surgery, Tata Memorial Hospital, Homi Bhabha National Institute, Mumbai, Maharashtra, India; 2 Department of Plastic Surgery, Kokilaben Dhirubhai Ambani Hospital, Mumbai, Maharashtra, India; 3 Department of Plastic and Reconstructive Surgery, Sir H. N. Reliance Foundation Hospital and Research Centre, Mumbai, Maharashtra, India

**Keywords:** back defects, posterior trunk defects, double perforator flaps, freestyle perforator flaps, lumbar, intercostal, paraspinal perforators

## Abstract

**Background:**

Large posterior trunk defects due to radical sarcoma excisions conventionally mandate a free flap, needing vein grafts for pedicle length and skin grafts for donor site. Conventional options like skin grafting or local (fasciocutaneous or myocutaneous) flaps are either unsuitable due to paucity of tissues or ill-advised in view of adjuvant radiotherapy. Perforator flaps are now an established option for back defects, and the use of single flap is quite common and widely reported. Larger defects can be dealt with by planning two such flaps on separate perforators.

**Materials and Methods:**

Retrospective analysis of consecutive double perforator flaps was done for indication of resection, size of defects, size of flaps, perforator origin, complications, and tolerance for radiation. Flaps were planned in freestyle manner, committed after visualization and dissection of the selected perforators through the defect, to enable best possible design for tissue recruitment and primary closure of donor sites.

**Results:**

Twenty-four flaps were performed in 12 patients. Average defect size was 168.5 cm ^2^ . One flap was lost to an arterial issue. Two flaps had venous insufficiency that resolved with release of sutures but needed secondary suturing and second flap respectively for marginal necrosis. Three cases needed skin grafts at remnant defects and site of suture dehiscence. Postoperative radiation was tolerated well.

**Conclusion:**

Double perforator flaps are viable alternative to free flaps for large posterior trunk defects. The native perforator-based supply and abundant skin of the back and neighboring trunk are well utilized to this effect. Primary donor site closure keeps morbidity to minimum.

## Introduction

Large complex defects over the posterior trunk are infrequently encountered secondary to cancer resections, trauma, spine surgeries, pressure sores, or congenital malformations. 1 2 3 4 5 6 These defects present a significant reconstructive challenge due to minimal local tissue laxity, paucity of recipient vessels for free flaps, and need of postoperative radiation.

Traditionally, muscle or musculocutaneous flaps have been used for posterior trunk defects, especially when vertebrae or orthopaedic hardware are exposed, as muscle is malleable enough to contour as well as cover and fill into dead spaces. 7 Typically available options are trapezius, latissimus dorsi, levator scapulae, rhomboideus, and paraspinal muscles as per the defect location. 3 They have limitations with regard to further function loss, arc of rotation, and defect size, hence, deemed inadequate or unfeasible for large sarcoma excisions.

Better understanding of the vascular anatomy of the back and perforator distribution has led to increasing use of local fasciocutaneous perforator-based flaps. It conceptually involves raising skin and subcutaneous tissues based on a single perforator vessel, which emerges from the underlying deep fascia and muscle. 7 Perforator flaps are now an established option to cover defects of the posterior trunk, but the use of a single flap is quite common and widely reported. Large surface area defects can be dealt with by planning two such flaps on separate perforators in vicinity of the defect.

This article reports a series of the freestyle double perforator propeller flap approach used to reconstruct the posterior trunk defects and evaluates its efficacy.

## Materials and Methods

Retrospective analysis of prospectively maintained data from departmental database and hospital electronic medical records for consecutive double perforator flaps between May 2006 and November 2016 was done. Twenty four perforator-based propeller flaps were performed in 12 patients for reconstruction of trunk defects secondary to excision of malignant tumors. Patient demographics, indication for resection, size and location of defects, size of flaps, perforator origin, and need for skin grafts were recorded. Outcome parameters were flap survival, wound complications, need of secondary procedures, and tolerance for radiation.

## Surgical Technique

Preoperative perforator mapping and flap planning are often rendered useless due to the vagaries and variability in the extent of resection. Hence, we practiced intraoperative assessment and planning in all cases. It was communicated to the primary surgeon to not undermine the wound edges during resection.

**Fig. 1:**
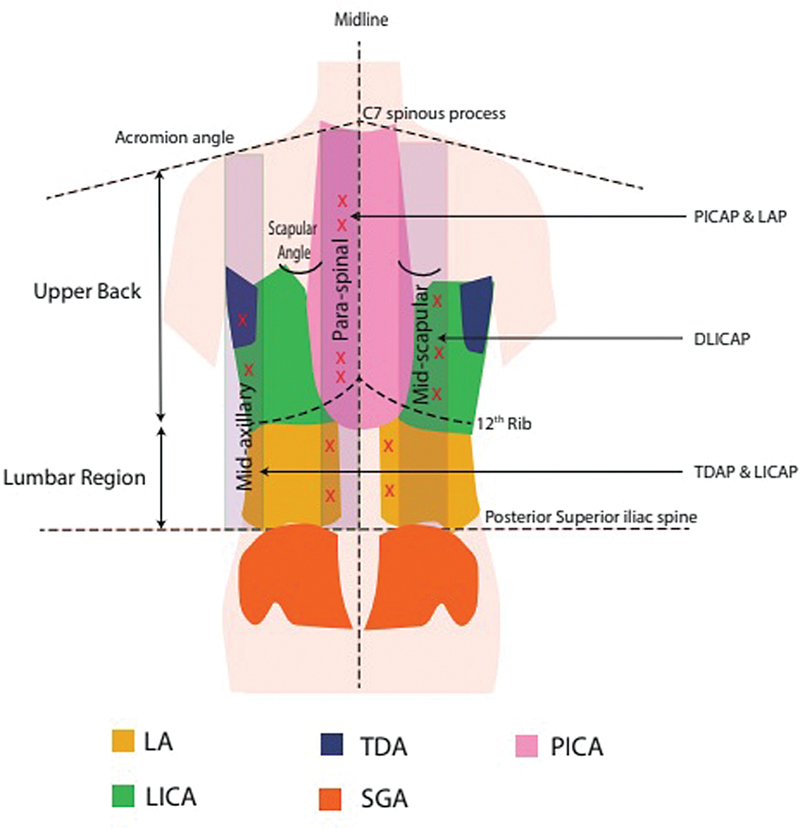
Diagrammatic representation of the surface marking of the perforators and their perforasome units. LA, lumbar artery; LICA, lateral intercostal artery; PICA, posterior intercostal artery (paraspinal); SGA, superior gluteal artery; TDA, thoracodorsal artery.

After wide excision of the lesion, perforator mapping was first done with a handheld Doppler up to 5 to 6 cm from the defect edges, taking into account the described surface markings for the perforators ( Fig. 1 ). Careful dissection was done through the wound to identify the perforator, reconfirming its size, adequacy, and integrity, and subsequently perforator selection and further dissection were conducted. In case of cut muscles (latissimus dorsi or serratus anterior), search of the perforator can be started deep to the muscle for surgical ease.

Perforator integrity and its precise entry point into dermis were confirmed again by handheld Doppler. Flaps were planned as freestyle ellipses around the isolated perforators to harvest best suited/available/pliable tissue to address the defect and enable primary closure of the donor sites avoiding skin grafts. Care was taken to keep the axes of the two flaps, 90 to 180 degrees to each other, in such a manner to not impede the donor closure of either flap. Flaps were raised from periphery to the perforator as a fasciocutaneous flap, and then transposed into the defect so as to have minimal, gentle torsion on the dissected perforator. The donor sites were closed first, as minor reorientation of defect happens. The flaps were secured in place with skin staples for approximately 30 minutes to unravel and assess any perfusion issues which might necessitate measures like further dissection of perforator, rotation of the flap in other direction, reducing the primary closure and placing minor skin grafts, and rarely delaying the flap transfer into the defect by a week. Small skin grafts at the tri-pointers are always a small price to pay compared to a tight skin closure risking compression of the perforator and flap necrosis.

## Results

Average age of the patients was 47.7 years (range: 11–64 years) with 10 males and 2 females. Histopathologically, the commonest etiology was soft tissue sarcoma (STS; 7 out of 12), followed by pleiomorphic spindle cell sarcoma (2), and dermatofibrosarcoma protuberans (2). Three patients had a recurrent tumor with history of previous excisions with primary closure, whereas nine were operated first time. Defect size varied from 11 × 8 cm to 25 × 14 cm with an average size of 168.5 cm ^2^ with the largest defect size covered was 350 cm ^2^ ( Table 1 ). Three defects were located in the upper thoracic, two in lumbar, and the rest seven involved both the regions of the posterior trunk. Most commonly used perforators were intercostal (16 of 24) and paraspinal perforators (6 of 24; Fig. 1 ). Average size of the perforators was 3 mm, and they were dissected through the muscles till the origin from the chest wall (intercostal space or the paraspinal muscles) yielding around 4 to 7 cm of length. The perforators located in the paraspinal region, posterior intercostal territory, and in the lower part of the trunk were more robust and consistent.

**Fig. 2:**
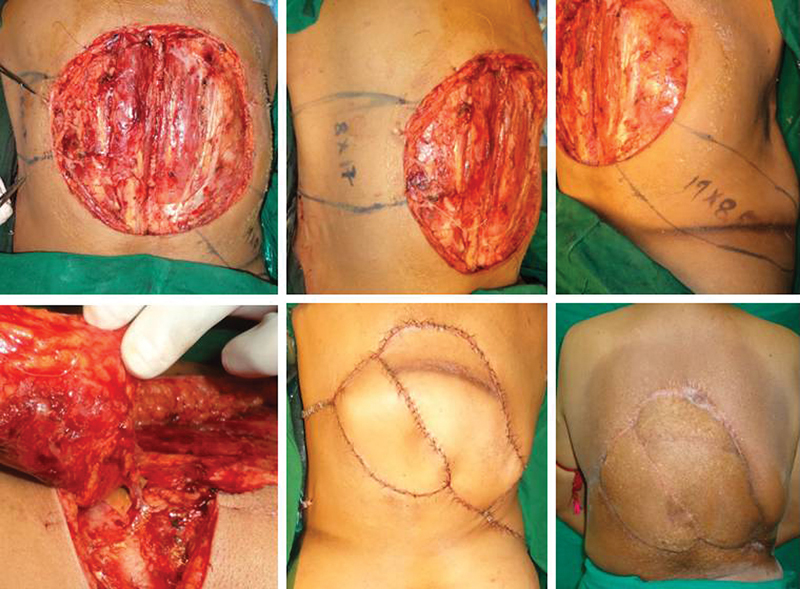
(Case 1) 20 × 14 cm defect post-excision of recurrent sarcoma, left (17 × 8) and right (19 × 8) sided LICA perforator propeller flaps planned and transposed for cover with primary donor-site closure with a post-radiation follow-up image. LICA, lateral intercostal artery.

One flap had arterial insufficiency and was lost. Two flaps had venous insufficiency in the early postoperative period, both resolved with release of sutures. They developed marginal necrosis requiring debridement and re-suturing in one and a perforator propeller flap in the other. Two cases needed small skin grafts at the residual defect and tri-pointer area. One donor site of a flap had a minor breakdown needing a small skin graft. All cases were followed up for a mean period of 20 months (range: 6–61 months). Ten patients needed postoperative radiation, who were followed up for a mean of 18.5 months, whereas other two had been previously radiated. There was no delay in radiation and it was tolerated without any complications ( Figs. 2 3 4 ).

**Fig. 3:**
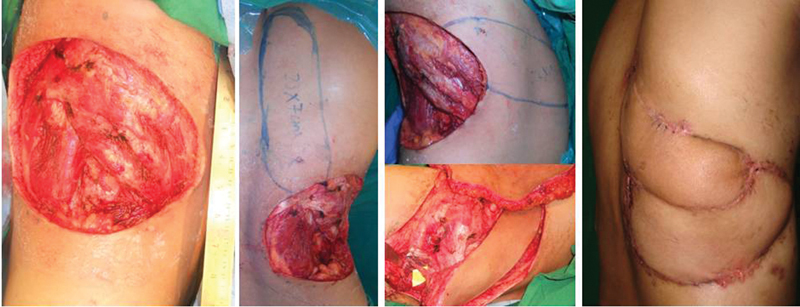
(Case 3) 25 × 14 cm defect post-excision of recurrent spindle cell sarcoma, left LICA (25 × 9) and right (23 × 7) sided paraspinal perforator propeller flaps planned and transposed for cover with primary donor-site closure. LICA, lateral intercostal artery.

**Fig. 4:**
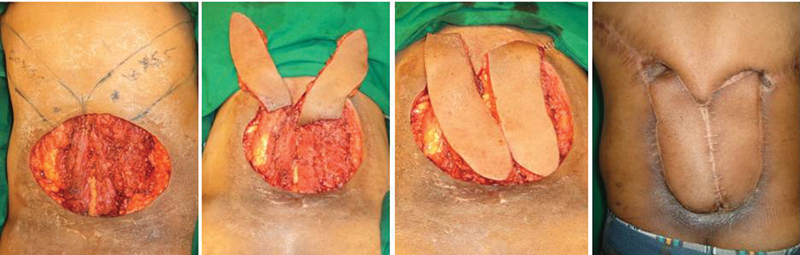
(Case 4) 20 × 12 cm defect post-excision of recurrent sarcoma, left (20 × 6.5) and right (20 × 6.5) sided paraspinal perforator propeller flaps planned and transposed for cover with donor sites closed primarily and small skin grafts, with a post-radiation follow-up image.

## Discussion

### The Problem

High-grade malignant lesions, like STS, often lead to large defects, which also need postoperative adjuvant radiation and are better served by well-vascularized tissues in the form of skin flaps. Perioperative radiotherapy also leads to local tissue ischemia, which compounds the skin graft and local flap-related morbidity if used. STSs are also prone to have multiple recurrences needing re-excision with extensive removal of tissues. This obviates the possibility of primary closure, depletes local muscle, and fasciocutaneous flap options. 8 9

### Conventional Options and Limitations

The ideal method for posterior trunk reconstruction should provide texturally similar and durable skin, robust vascularity, easy, versatile, and reproducible technique, and minimal aesthetic or functional donor-site morbidity. Traditionally described options for such defects are primary closure, wide undermining with skin advancement, split skin graft, local flaps including various rotation, advancement or transposition flaps, bilobed flaps, Z-plasties or lumbosacral fasciocutaneous flaps, transposed muscle flaps using latissimus dorsi, gluteal, trapezius or paraspinous muscles with split skin graft, musculocutaneous flaps 10 with split skin grafting of secondary defect, perforator-based flaps with either a propeller or a keystone design, and free flaps.

Local fasciocutaneous flaps are often not an option for such large defects due to tissue inadequacy, especially in Indian context where people generally have low body mass index with limited tissue pliability, which leads to limitation in planning liberties. Although muscle flaps can fill and cover irregular cavities, 11 the obvious disadvantages of such flaps are fixed pivot points and poor reach at mid and lower back, using the distal most part of the flap or on secondary supply with unreliable vascularity, donor morbidity, and a highly possible sacrifice in extensive resections which makes them unavailable for use in reconstruction.

Free flaps can be used for reconstruction anywhere on the body, and can match the defect qualitatively as well as quantitatively. Increased technical difficulty, paucity of available recipient vessels to support the microvascular transfer, and application of alternative methods, such as arteriovenous looping and interposition vein grafts with higher complication rates, make this a challenging option. 12 13 14 Due to the large size of flaps, it needs skin grafting for donor-site cover, leading to additional morbidity. 15 The intra-operative position changes for a free flap prolong the surgical time and positioning and immobilization needed in the postoperative period compound the difficulty in this reconstructive approach. These limitations have led to exploration and application of perforator flaps in back reconstruction.

### The Solution—Perforator Flaps

After initial introduction of perforator flap in 1989, there was a rapid increase in the use of perforator flaps with advantages of sparing of the underlying muscle with resultant decreased donor-site morbidity. 16 Availability of a substantial number of perforators in the body provides the surgeons a versatile and non-microvascular simpler reconstructive alternative. 17

The posterior trunk can be described as the region between the line joining the spinous process of C7 bilaterally with the acromial angle to the line joining the two posterior-superior iliac spines and extending laterally to mid-axillary lines. 18 This can be further subdivided into upper back and lumbar regions by the lower border of 12th rib.

The upper back region has around 24 perforators with corresponding distinct perforasomes, superiorly from branches of thyrocervical trunk, laterally from the subscapular axis, medially from the paraspinal perforators, and laterally from the lateral branches from posterior intercostal arteries. 18 The lumbar region is supplied by robust and large perforators from four paired sets of lumbar arteries, whereas the gluteal region has numerous musculocutaneous perforators from superior and inferior gluteal arteries ( Fig. 1 ). 18

These perforasomes have large overlapping territories, thus allowing the freestyle approach, to plan pedicled propeller flaps for defects involving all regions of the posterior trunk. Several authors report the clinical reliability and successful application of this approach. 19 20

Various perforator flaps that can be planned on these perforators to cover posterior and lateral torso defects are posterior intercostal artery perforator flap or paraspinal perforator flap, dorsolateral intercostal artery perforator flap, lateral intercostal artery perforator (LICAP), anterior intercostal artery perforator or internal mammary perforator flap, lumbar artery perforator flap, and superior gluteal artery perforator flap. 21 22 Perforator options in the posterior trunk region in the order of utility are intercostal, paraspinal, and lumbar, which are the most commonly used perforators in our series as well. Detailed anatomical description with mapping of the cutaneous territories, size of perforators, and the distribution of perforators has been well described.

Due to their widely reported and obvious advantages over conventional methods, perforator-based flaps are now established as a first-line reconstructive option for moderate to large-sized back defects with commonly used designs being propeller or keystone. Keystone design perforator islanded flaps are now increasingly used on account of technical simplicity over propeller design as localization and dissection of perforators is not necessary and limited undermining to facilitate the flap movement into the defect.

The back skin is stiffer and thicker owing to more dermis, with a nonpliable subcutaneous tissue. This proves a major hurdle in local flaps and keystone flaps, which principally advance into the defect. It may lead to closure under tension with the suture line formed by the defect edges, which may be thinned or irradiated causing wound healing issues. In contrast, propeller design proves to be more in line with the principle of tissue recruitment from an adjacent area with more laxity into the defect without surrounding laxity. This creates a secondary defect which can be closed primarily.

Dorsal intercostal artery perforators or paraspinal perforators are usually found 5 cm lateral to the midline from 3rd to 11th thoracic vertebrae ( Fig. 1 ). 23 24 Flaps based on these perforators for posterior midline defects were first reported in 1988 and are now widely utilized for posterior trunk coverage. Skin territories extending to mid-axillary line and iliac crest inferiorly (lower perforators) can be safely raised on these perforators and the largest reported flap is 40 × 15. 24

Intercostal perforators prove to be the most reliable and go to option as extensive and freestyle skin paddles can be planned on them as intercostal perforators are reported to perfuse their own angiosome as well as more than two adjacent perforasomes on account of linking true anastomoses with the same caliber between adjacent territories. 22 25 They have widely distributed cutaneous perforators along paramedian region, midscapular line, and mid-axillary lines. All these perforator branches and arborization converge on the lateral flank region and have significant deep and superficial anastomoses, which provide numerous options and versatility in designing for possible flap elevation. Badran et al have reported flaps as large as 25 × 20 cm based on LICAPs located at the mid-axillary line. 26 27 Arco et al have reported maximum coverage of 264 cm ^2^ by using a single flap, whereas the largest flap in our series was 25 × 9 cm (225 cm ^2^ ) based on LICAP. 28

However, in extensive sarcoma resections, a single perforator flap is seldom sufficient and the commonly utilized approach is to either cover the critical area with the flap and skin graft the rest or use a free flap for cover. Also, two separate perforator-based flaps can be effectively planned and executed for defect coverage. This principally avoids the morbidity related to the free flaps or skin grafts and provides the benefits of locoregional perforator flaps. Free flaps can be reserved as salvage or secondary option. de Weerd and Weum have described a “butterfly design” of two pedicled perforator flaps for covering sacral pressure sore. 29 We report the largest series of double perforator propeller flaps for coverage of extensive back defects post-malignancy excision.

However, this approach to back defects has to be employed with due caution. Execution of these flaps requires careful and diligent planning for flap size, axis, and transfer. Meticulous and skillful dissection of the perforator is imperative when it is being cored out with or without the muscle cuff that helps support a delicate perforator, which sometimes makes it a tedious and time-consuming procedure. It is also quite difficult to predict perforator to flap size ratio, i.e., what size of flap a given size of perforator will sustain. There are no established guidelines in literature as well for this. Hence, there is a theoretical risk of marginal necrosis, secondary procedures, and associated morbidity with delayed adjuvant radiotherapy.

## Conclusion

Double freestyle perforator flaps are a viable, suitable, and reproducible alternative to free flaps for large posterior trunk defects post-oncological ablative surgery. The native perforator-based blood supply and abundant skin of the back and neighboring abdomen are well utilized to this effect. Primary closure of the donor site keeps donor morbidity to minimum. Our results underline the versatility and safety of this approach and expand the indications of perforator flap-based trunk reconstruction to extensive defects as well.

**Table 1:** Summary of operated patients

Case no.	Age (y)	Sex	Histopathology	Primary/recurrence (pre-op RT +/−)	Size of defect (in cm)	First flap (in cm)	Perforator used (source vessel) [size in cm]	Second flap (in cm)	Perforator used (source vessel) [size in cm]
1	48	F	STS	P (−)	20 × 14	17 × 8	Lt LICA [3]	19 × 8	Rt LICA [2.5]
2	57	M	DFSP	R (−)	17 × 13	17 × 7	Lt PICA [3.5]	15 × 6	Rt paraspinal [3.5]
3	33	M	STS	R (−)	25 × 14	25 × 9	Rt LICA [3]	23 × 7	Rt paraspinal [3.5]
4	64	M	Pleomorphic spindle cell sarcoma	P (+)	20 × 12	20 × 6.5	Lt paraspinal [3]	20 × 6.5	Rt paraspinal [3]
5	43	M	Leiomyosarcoma	R—4th surgery (−)	20 × 10	20 × 8	Rt LICA [2.5]	17 × 6	Rt IMAP (4th) [2.5]
6	42	M	STS	P (−)	11 × 8	10 × 5	Lt PICA [3.5]	10 × 5	Rt PICA [2.5]
7	45	M	STS	P (−)	15 × 8	15 × 5	Lt LICA [2]	14 × 4	Rt PICA [2.5]
8	11	M	STS	P (−)	25 × 10	15 × 5	Lt Lumbar [2.5]	15 × 5	Rt PICA [3]
9	28	M	DFSP	P (−)	22 × 12	20 × 7.5	Lt PICA [4]	20 × 7	Rt LICA [3.5]
10	64	M	Pleomorphic spindle cell sarcoma	P (+)	18 × 10	18 × 5	Lt PICA [3.5]	17 × 4	Rt paraspinal [3.5]
11	39	F	STS	P (−)	14 × 11	12 × 6	Lt paraspinal [3]	12 × 7	Lt PICA [2.5]
12	44	M	STS	P (−)	18 × 16	18 × 7	Lt PICA [3]	17 × 7	Rt PICA [3.5]

Abbreviations: DFSP, dermatofibrosarcoma protuberans; IMA, internal mammary artery; LICA, lateral intercostal artery; PICA, posterior intercostal artery; STS, soft tissue sarcoma.
